# The MicroRNA Family Both in Normal Development and in Different Diseases: The miR-17-92 Cluster

**DOI:** 10.1155/2019/9450240

**Published:** 2019-02-03

**Authors:** Xiaodan Bai, Shengyu Hua, Junping Zhang, Shixin Xu

**Affiliations:** ^1^First Teaching Hospital of Tianjin University of Traditional Chinese Medicine, Tianjin 300193, China; ^2^Tianjin Key Laboratory of Traditional Research of TCM Prescription and Syndrome, Tianjin 300193, China; ^3^Tianjin University of Traditional Chinese Medicine, Tianjin 300193, China; ^4^Department of Cardiology Medicine, First Teaching Hospital of Tianjin University of Traditional Chinese Medicine, Tianjin 300193, China

## Abstract

An increasing number of research studies over recent years have focused on the function of microRNA (miRNA) molecules which have unique characteristics in terms of structure and function. They represent a class of endogenous noncoding single-strand small molecules. An abundance of miRNA clusters has been found in the genomes of various organisms often located in a polycistron. The miR-17-92 family is among the most famous miRNAs and has been identified as an oncogene. The functions of this cluster, together with the seven individual molecules that it comprises, are most related to cancers, so it would not be surprising that they are considered to have involvement in the development of tumors. The miR-17-92 cluster is therefore expected not only to be a tumor marker, but also to perform an important role in the early diagnosis of those diseases and possibly also be a target for tumor biotherapy. The miR-17-92 cluster affects the development of disease by regulating many related cellular processes and multiple target genes. Interestingly, it also has important roles that cannot be ignored in disease of the nervous system and circulation and modulates the growth and development of bone. Therefore, it provides new opportunities for disease prevention, clinical diagnosis, prognosis, and targeted therapy. Here we review the role of the miR-17-92 cluster that has received little attention in relation to neurological diseases, cardiac diseases, and the development of bone and tumors.

## 1. Introduction

miRNAs constitute a class of endogenous noncoding single-strand small molecules which are highly conserved. Their discovery expanded our understanding of gene expression regulation mediated by RNA, revealing a substantially more intricate system than previously hypothesized. Recent reports demonstrate that novel and highly significant breakthroughs in the fields of disease diagnosis, treatment, and biomarkers based on miRNAs have been established, starting a new in-depth study of miRNAs. miRNAs are generally 20~25 nucleotides in length and combine with the target gene at the 3' untranslated region (UTR) by complete and/or partial complementarity, thereby regulating gene expression. miRNAs can also directly cut or degrade the target gene messenger RNA (mRNA) and inhibit the translation of the mRNA.

It has been established for some years that the miR-17-92 gene cluster is oncogenic, playing a role in various cancers. However, as research has progressed, it has been found that it causes tumors, and also it has been found increasingly to be responsible for other diseases and their regulation, for instance, strokes. Induction of the regeneration of neural tissue after an ischemic stroke and nerve injury is achieved mainly through two different methods, on one hand, the induction of endogenous neural stem cell regeneration and, conversely, the transplantation of exogenous stem cells. Although the induction of endogenous stem cell regeneration avoids the risk of transplantation and has made some progress, satisfactory therapeutic levels have not yet been achieved. Stem cell transplantation is a research subject undergoing intense study in the field of nerve regeneration. The study of mesenchymal stem cells (MSCs) is very comprehensive, but, nevertheless, transplantation of MSCs also faces the risk of brain tissue damage and infection for targeted transplantation, in addition to issues regarding the efficiency of transplantation for intravenous infusion. Exosomes are microvesicles secreted by cells with a diameter of approximately 30-100 nm, containing a large number of biologically active substances such as protein molecules, DNAs, mRNAs, and miRNAs, which permits intercellular communication through noncellular contact. Exosomes from MSCs were first reported in 2010 in a myocardial ischemia-reperfusion study of mice that attempted reduction in myocardial infarction [1]. Since then, several studies have shown that MSCs mediate cell communication by releasing a large number of exosomes, thereby regulating endogenous damage repair of brain tissue [2–5]. The expression of target gene networks in effector cells via miRNAs is an important mechanism by which MSCs-exosomes perform a biological role. The miR-17-92 gene cluster regulates the proliferation and differentiation of nerve cells. In the MCAO mouse model with permanent infarction, the levels of miR-17-92 clusters in neural progenitor cells from the subventricular zones (SVZ) were found to be increased significantly, where they regulated neural progenitor cell proliferation [6]. During the normal development of the mouse cerebral cortex, the miR-17-92 cluster regulates the proliferation and differentiation of neural stem cells in the cortex to form intermediate progenitor cells through the inhibition of the expression of the downstream target gene PTEN [7]. Axonal regeneration disorder is a key issue affecting the recovery of neurological function after central nervous system injury. Research studies have confirmed that miR-17-92/PTEN/PI3K/Akt/mTOR is a vital pathway regulating the growth of neurons and axonal regeneration [8].

In addition to this evidence, the miR-17-92 cluster has recently been shown to perform a critical role in the development of heart tissue [9–11] (Table 1) and bone [12] (Table 1). Although these pioneer studies have been guiding current miR-17-92 cluster research, pointing to its important role in many diseases, a lack of comprehensive analysis or understanding of its biological functions hinders progress in the miR-17-92 cluster research. It is worth that as in the past, and prior to progress in any new technology, researchers are at the stage of describing the different sources and components of the miR-17-92 cluster and are beginning to analyze the genome. The well-known roles of miRNAs and the theories regarding endogenous RNAs have expanded the research horizons of the miR-17-92 cluster.

## 2. The Unique Polycistronic Structure of the miR-17-92 Cluster

miRNAs are small noncoding RNAs that direct posttranscriptional gene silence in eukaryotes. Their coding is often clustered in the genomes of animals and can be independently transcribed or simultaneously into a single polycistronic transcript [13, 14]. In most cases, clustered miRNAs belong to the same family, forming homologous clusters. These miRNAs have the same “seed region,” resulting in functional redundancy, but the published miRNAs research appears contradictory [15]. Polycistronic miRNA genes have different forms, some of which comprise homologous miRNA components [16–18], some are composed of nonhomologous components, and some contain both homologous and nonhomologous miRNA components [17]. Some clusters of miRNA genes are transcribed by a common promoter to generate a polycistronic transcript, which is then processed to generate multiple mature miRNAs. The miR-17-92 cluster is a representative example of a polycistronic miRNA gene [19]. It is located in the 13q31.3 region of human chromosome 13 and produces seven individual mature miRNAs, miR-17-3p, miR-17-5p, miR-18a, miR-19a, miR-20a, miR-19b, and miR-92a [17, 19–22]. It has two mammalian paralogs, the miR-106b-25 (located on human chromosome 7) and miR-106a-363 clusters (located on the X chromosome). The miR-106b-25 cluster encodes miR-106b, miR-93, and miR-25; the miR-106a-363 cluster encodes miR-106a, miR-18b, miR-20b, miR-19b-2, miR-92a-2, and miR-363 [19, 21, 23, 24]. When miRNAs recognize their target genes and exert posttranscriptional regulation, it is nucleotides 2-8 of miRNAs that participate in the recognition events, i.e., their seed region sequences. Therefore, we divided the miR-17-92 gene cluster and its homologous genes into four families based on their seed sequences, the miR-17 family (miR-17, miR-20a/miR-20b, miR-106a/miR-106b, and miR-93), the miR-18 family (miR-18a/miR-18b), the miR-19 family (miR-19a/miR-19b), and the miR-25 family (miR-25, miR-92a, and miR-363). Members of the miRNA gene cluster cooperate in the regulation of certain processes or play an associated role in the same biological process, so that multiple members perform their functions more effectively and ensure normal and orderly biological activity [25], Figure 2. Thus regulation of overlapping or complementary target groups induces biological responses [26]. A large number of miRNAs are located in polycistronic miRNA clusters, where multiple miRNA genes are produced by a single primary transcript [21, 26, 27]. Studies demonstrate that approximately 50% of* Drosophila melanogaster* and more than one-third of human miRNA genes are clustered together. The highly conserved nature of miRNA clusters across species illustrates the high evolutionary pressure for maintenance of this form of genomic organization [21]. In addition, recent genome-wide mapping of Kaposi's sarcoma-associated herpesvirus (KSHV) and its 3'UTRs has indicated large bicistronic and polycistronic transcripts. The 3'UTRs extensions of the 5' terminal gene of bicistronic and polycistronic transcripts provide additional regulatory targets [28]. Some findings have extended our understanding of the uniqueness of KSHV gene regulation and provide valuable resources for the study of other genomic processes. The balance of polycistronic microRNA is regulated in a hierarchical manner after transcription, which may involve ecotropic virus integration site 1 (EVI1) [29]. The variations in organization of miRNA genes which acts as polycistronic transcripts clarify a multifunctional mechanism that controls gene expression in different biological processes [13]. The existence of miRNA gene clusters has greater significance than single miRNA genes in the understanding of functional and evolutionary relationships. It has been reported that fewer than half of the genes so far identified are expressed in polycistronic clusters [26].

## 3. Biological Function of the miR-17-92 Cluster

miRNAs participate in various basic cellular functions through posttranscriptional regulation of target genes [30]. The miR-17-92 cluster is highly expressed in embryonic cells [16, 31, 32] but decreases following terminal differentiation [19]. The maturity level of each member of the miR-17-92 cluster may vary in different cell types [33]. Newborns generally die soon after birth if the miR-17-92 cluster is eliminated in the early stages of fetal development [12, 34]. It has been postulated that the miR-17-92 cluster modulates neurogenesis and angiogenesis. It regulates the proliferation of neural progenitor cells both during development and in nervous system diseases such as following a stroke [35, 36]. Evidence also suggests that the endothelial miR-17-92 cluster regulates angiogenesis during the embryonic period and adulthood [36] (Table 1). The distal axons of the neurons express the miR-17-92 cluster which regulates axonal outgrowth. The local modulation of PTEN protein levels by miR-19a may promote oligodendrogenesis, neurogenesis [37–39], and axonal outgrowth [8, 37]. miRNAs are indispensable in the occurrence and development of cerebral ischemia in addition to postischemic vascular repair [40], modulating the process. Also important are the Notch and sonic hedgehog (Shh) signaling pathways in mediating stroke-induced neurogenesis which involves the interactions between miR-124 and the miR-17-92 cluster [38]. Additionally, the lack of circulating miRNAs in peripheral blood following cerebral ischemia is considered to be a meaningful biomarker for cerebral ischemia additional to cardiovascular system disease diagnosis and prognosis [11, 30, 41–43]. Individual miRNAs that belong to the miRNAs-17 family differ in their consequences during differentiation of embryonic stem cells [6]. The miR-17-92 cluster and its paralog miR-106b-25 perform an important function in the cardiovascular system by mediating the process of angiogenesis. It has been established that miR-106a, miR-17, and miR-93 target the Friend of Gata-2 (Fog2) gene in the cardiac suppressor, which specifically suppress Gata-4 and Coup-TF2. However, it has been observed that the differential target efficacies for Fog2 lead to varying degrees of cardiac differentiation as each miRNA becomes knocked out [44]. Furthermore, miR-17-92 is regarded as a one of the “cardiometabolic miRs” which are thought to reside in the circulation, participating in the development of cardiovascular disorders such as coronary artery disease (CAD) and cardiac ischemia/reperfusion (I/R) injury in addition to related cardiovascular risk factors; it is also considered as a metabolic and cardiovascular diseases' biomarker [45–49]. Other evidence has suggested that miR-92 is involved in dilated cardiomyopathy and myocardial ischemia [50, 51]; it is a latent target for innovative therapeutic tactics to prevent cardiac remodeling [51]. Interestingly, it is possible that the two paralog clusters such as miR-106a~miR-363 and miR-106b~miR-25 originate from sequence duplication and deletion during the evolution of vertebrates [15]. A number of studies demonstrate that absence of the miR-17-92 cluster is the fatal issue during the perinatal stage, miR-17-92 deletion at the time of embryos causes severe skeletal abnormalities [12, 38], and the sizes of experimental mice are also smaller compared with normal embryos, dying at birth due to cardiac defects and lung hypoplasia [16]. The miR-17-92 cluster together with its two paralogs 106a-363 and 106b-25 is thought to exhibit oncogenic properties which are constantly upregulated in diverse established osteosarcoma (OS) cell lines. Wherefore we stress a major role in the pathogenetic and prognostic impact of the miR-17-92 cluster and its paralogs in OS biology [52]. Additionally, other reports have shown the widespread utilization of the miR-17-92 cluster: miR-17 can improve inflammation-induced insulin resistance by inhibiting the expression of apoptosis signal-regulating kinase 1 (ASK1) in macrophages. Results confirmed that miR-17 has an anti-inflammatory effect on macrophages, thereby exhibiting antidiabetic activity [53]. Targeted suppression of the miR-17-92 cluster resulted in severe atrophy of testes in adult mice and increased apoptosis of germ cells in testes of miR-17-92 deficient mice [34]. Deletion of miR-17-92 is lethal postnatally with multiple developmental defects, including lung hypoplasia and ventricular septal defects. Some patients also exhibit varying degrees of learning and developmental disabilities.

## 4. Target Gene of the miR-17-92 Cluster

miRNAs operate by modulating the expression of numerous target genes [54, 55], although the target genes are differentially sensitive to inhibition by miRNAs, and only a small proportion are inhibited by miRNAs under physiological conditions. miRNA-17-92 probably regulates the expression of target genes through translational repression, and the 5'UTR regulates the sensitivity of target genes to miRNA inhibition. In short, the current hypothesis is that miRNAs exert their specific function via a small section of specific target genes rather than several genes [54]. More than 30 downstream target genes of the miR-17-92 gene cluster have been found, such as the amyloid precursor protein (APP) gene, cyclin D1 (CCND1), TBC1D2/Armus, transcription factor E2F, mitogen-activated protein kinase 9 (MAPK9), and large tumor suppressor kinase 2 (LATS2) [56]. Among them, the apoptotic gene BCL2L11 (Bim) [16, 56], human interferon regulatory factor (IRF), c-Jun amino terminal kinase 2 (JNK2)/mitogen-activated protein kinase 9 (MAPK9), MYCN, pathogenetic gene 2 (PKD2) of polycystic kidney disease, Grb2 anchoring protein 1 (GAB1), rhamnolipid 1 (RBL1), tumor sensitive gene 101 (TSG101), p63, signal transduction, signal transduction transcriptional activator 3 (STAT3) [34], p38/MAPK14, transforming growth factor-*β* (TGF-*β*), hypoxia inducible factor-1*α* (HIF-1*α*), Rbl2/pl30, p57, p27 [56], and p21 [56, 57] are involved in the regulation of cell cycle in tumors [56].

Shh is a member of the hedgehog protein family and plays a role in modulating proliferation and differentiation in brain neural progenitor cells [38, 58]. Accordingly, it is logical that modulation of the Shh pathway produces a marked effect in stroke-induced neurogenesis [38]. Neural progenitor cells can be observed in the subventricular zone (SVZ) after cerebral ischemia if the miR-17-cluster is upregulated. Overexpression of the miR-17-92 cluster together with miR-18a and miR-19a enhances stroke-induced progenitor cell proliferation; the active consequences of the miR-17-92 cluster on neurogenesis may be ascribed to the reduction in the phosphatase and tensin homolog deleted on chromosome 10, which negatively regulates embryonic neural stem cell proliferation and survival [6, 38].

It has been reported that the miR-17-92 cluster acts positively in cardiac development and remodeling. CTGF and TSP-1 have been considered as target genes of miR-18a and miR-19a. Consistent with these findings, miR-18a and miR-19a were observed to be clearly inhibited in older cardiomyocytes, while the expression levels of CTGF and TSP-1 had increased [59], demonstrating the close relationship of miR-18a and miR-19a to CTGF and TSP-1. Overexpression of miR-92a inhibits the apoptosis of vascular smooth muscle cells induced by H_2_O_2_ through the immediate targeting of the mitogen-activated protein kinase 4-c-Jun N-terminal kinase 1 (MKK4-JNK1) pathway [60].

Studies have verified that the miR-17 family participates in bone morphogenetic protein (BMP) signaling, which is positive in many tissues such as the central nervous system, in which it is principally responsible for controlling cell proliferation, differentiation, and maturation. In line with these findings, we have observed that BMP2 stimulation directly enhances miR-17-92 and miR-106b-25 cluster transcription due to the activation of Smad, resulting in the upregulation of mature miR-17/miR-20a/miR-93. Additionally, BMP2 activation can repress bone morphogenetic protein receptor II (BMPRII) expression by regulating the miR-17 family in primary neurons. Furthermore, abnormal BMP signaling results in the apoptosis of neurons which can be circumvented by negative regulation. Overall, the regulation of the BMP-miR-17 family-BMPRII pathway that comprises a negative feedback loop can balance BMP signaling and maintain homeostasis in neurons [61].

Since it has been established that PTEN is a specific target of miR-19a [7], PTEN and E2FS have been confirmed as miR-17-92 targets [16]. There are data showing a miRNA dynamically regulates CD8 T-cell differentiation from the naive stage to effector and memory phases, with short-lived effector cells expressing oncogenic miR-17-92 to high level, compared with the slower proliferating memory-fated cells [62]. A number of studies concluded that miR-17-92 regulates proliferation and survival via reduction in the expression of PTEN, a negatively regulating molecule, thereby lifting the suppression of the PI3K/AKT/mTOR signaling pathway. Faster acquisition of memory properties emerges as miR-17-92 levels are reduced. It is beneficial, therefore, to control miR-17-92 expression during immunization in order to optimize T-cell memory quantity and quality [62]. Increased proliferation upon miR-17-92 overexpression correlated with decreased expression of the tumor suppressor PTEN and increased PI3K AKT/mTOR signaling [62].

A study that conduct a series of predictions and analyses confirmed that PTEN, a tumor suppressor, is revealed to be a bona fide target of both miR-92a and miR-19a in cholangiocarcinoma. It has been reported that other targets of the miR-17-92 cluster exist in human cholangiocarcinoma cells, APAF-1 and PRDM2 included. In addition, IL-6/Stat3, a carcinogenic signaling pathway key to the manifestation of cholangiocarcinoma, regulates the expression of the miR-17-92 cluster [22], which boosts cell proliferation and survival of B-lymphoma cell lines and primary tumors by targeting CDKN1A and PTEN. In summary, miR-17-92 is a downstream effector of fibroblast growth factor receptor 1 (FGFR1) in BCR-FGFR1-driven B-cell lymphoblastic leukemia [63].

miR-17-92 is an effective inhibitor of transforming growth factor- (TGF-) *β* signaling [64]; MYC is a transcription factor that upregulates the expression of miR-17-92 in neuroblasts, with p21 also a target gene of miR-17-92. Its expression is inhibited by C-MYC, which triggers the proliferation of tumor cells and eventually leads to the development of tumors. miR-20 influences activation of the TGF-*β* regulatory factor CDKN1A/p21, thereby preventing the antiproliferative effect of TGF-*β* in colorectal cancer.

## 5. Effect of the miR-17-92 Cluster on Disease States

### 5.1. miR-17-92 Cluster in Neurological Diseases

Stroke has become a leading cause of mortality and disability worldwide due to its numerous pathogenic factors and lack of treatments. A large number of recent studies investigating miRNAs support their central role in ischemic diseases. Evidence confirms that miRNAs are a particularly important choice for stroke therapy [65] because they regulate many target genes simultaneously while therapeutic interventions against single genes have not so far been successful. There have been many discoveries of miRNAs in ischemic heart disease, but less is known about their role in cerebral ischemia [66]. Here, we discuss the miR-17-92 cluster, as a novel factor in strokes.

Cerebral ischemia causes neurogenesis, including neural progenitor cell proliferation and differentiation in addition to the migration of freshly recently produced neuroblasts. Emerging evidence makes it worth believing that miRNAs may perform a vital role in mediating adult neural progenitor cell proliferation and differentiation [67] (Table 1).

Research studies have demonstrated that different concentrations of the miR-17-92 cluster in adult hippocampal neural progenitor cells promote neurogenesis and anxiety and depression-related behaviors in mice. Mice show anxiety- and depression-like behavior when miR-17-92 is knocked out compared with those that exhibit anxiolytic and antidepression-like behaviors when overexpressing miR-17-92. miR-17-92 influences neurogenesis by regulating genes such as serum- and glucocorticoid-inducible protein kinase 1 (Sgk1) in the glucocorticoid pathway [68] (Table 1). Of note is the observation that the miR-17-92 cluster is in abundance within in embryonic stem cell- (ESC-) derived limb-innervating lateral motor column motor neurons (LMC-MNs), and its deletion in MNs leads to LMC-MNs dying both* in vitro* and* in vivo.* miR-17-92 cluster overexpression prevents the apoptosis of MNs, which occurs spontaneously during embryonic development. PTEN is the main target of miR-17-92 clusters which control the degradation of LMC-MNs. This miRNAs-mediated regulation modulates the expression of the target and its subcellular localization, causing LMC-MNs to have complex mechanisms that control their command survival [32].

**Figure 1:**
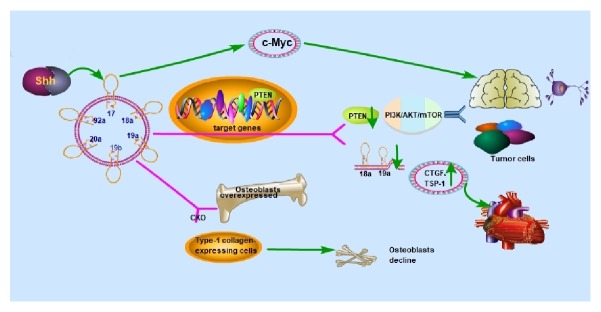
The miR-17-92 cluster participates in the development of diverse diseases by regulating the common target gene, contributing to neurological plasticity, functional recovery, and development of tumors via activation of the PI3K/AKT (protein kinase B)/mTOR (mammalian target of rapamycin) PI3K/AKT/mTOR pathway by downregulation of the phosphatase and tensin homolog (PTEN). A decrease in miR-18a and miR-19a levels leads to an increase in connective tissue growth factor (CTGF) and thrombospondin-1 (TSP-1). They are considered to be differentially expressed in relation to the many cardiac miRNAs in cardiomyocytes developing. The cluster is also essential for the development and maintenance of bone strength.

**Figure 2:**
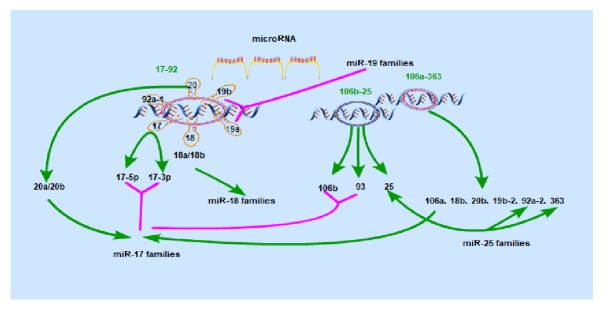
Structure of the miR-17-92 polycistronic gene. Some miRNA genes are clustered on chromosomes, transcribed by a common promoter to generate a polycistronic transcript, and then processed to produce multiple mature miRNAs. In some miRNA gene clusters each member has a functional additive effect that is more effective than a single miRNA. Members of the miRNA gene cluster can coordinate the regulation of certain processes or play a similar role in the same biological process, ensuring biological activity occurs in a normal and orderly fashion. miR-17-92 encodes a miRNA precursor and produces 7 mature miRNA molecules that belong to 4 miRNA families.

Treatment of strokes with exosomes containing trimmed miR-17-92 clusters contributes to neurological plasticity and functional recovery, probably through activation of the PI3K by targeting PTEN [37, 69] (Figure 1) (Table 1).

### 5.2. The miR-17-92 Cluster in Heart Diseases

The development of the heart is an astonishing process which involves the differentiation of cardiac precursors into a number of cell types including the myocardium, cardiac mesenchyme, and endocardium. Each class cell contributes a particular program to the formation of the cardiac chambers, conduction system, and highly specialized valvuloseptal structures. It is apparent that the precise modulation of the development of the heart is essential for its normal function [70].

Irrespective of the role that the miRNA families perform in the development of the heart, they certainly take part in the regulation of the fate of cardiomyocytes [71]. For example, miR-133a that is expressed in cardiac hypertrophy [72, 73] is promoted by adiponectin which decreases extracellular signal-regulated kinase (ERK1/ERK2) phosphorylation through AMP-activated protein kinase (AMPK) activation [72]. Conversely, the miR 30-CSE-H_2_S axis prevents myocardial cell ischemic injury (MI) by regulating the production of hydrogen sulfide (H_2_S). miR-30 family repression after MI provides potential therapeutic value for the normal functioning of the heart [74, 75]. The endoplasmic reticulum (ER) is a membranous and multifunctional organelle that is involved in many processes in the majority of animal cells, cardiomyocytes and vascular cells included. ER stress is identified as a prerequisite in the initiation and development of cardiovascular diseases, including heart failure, ischemic heart disease, diabetic cardiomyopathy, atherosclerosis, hypertension, and strokes. The core of ER stress is an increase in the expression of glucose regulatory protein 78 (GRP78) which is known to be one of the target gene for miR-30. Researchers have speculated that the downregulation of the miR-30 family induces ER stress and related upregulation of GRP78 in the cardiovascular system, as the involvement of miR-30 produces a positive effect toward ER stress. miR-30 has proved to be a viable method of alleviating diseases connected with ER stress [75]. Notably, the miR-17-92 cluster is considered to be differentially expressed in cardiomyocytes at various developmental stages, but, among the many cardiac miRNAs, little is known about whether miR-17-92 provides regulatory mechanisms in heart disease (Figure 1). Of late, it has been confirmed that the miR-17-92 cluster is a key regulator of cardiac proliferation. Transgenic overexpression of the miR-17-92 cluster causes cardiomyocyte proliferation in embryonic, postnatal, and even adult hearts via inhibition of PTEN. Furthermore, miR-17-92 cluster overexpression reverses cardiac dysfunction and cardiac fibrosis following myocardial infarction. Thus, it is believable that the miR-17-92 cluster promotes both neurogenesis and angiogenesis and acts as a target in CNS regeneration therapies [36]. Moreover, miR-17-92 clusters are involved in the differentiation of monocytes to macrophages and activation of macrophages, which have a pivotal role in the physiological and pathophysiological process of atherosclerosis. The miR-17-92 cluster and HDAC9 gene participate in processes related to unstable carotid plaques activities such as inflammation, apoptosis, and angiogenesis [76]. Previous studies of endothelial cells indicate that HDAC9 promotes angiogenesis by inhibiting the miR-17-92 cluster [76] (Table 1).

The differentiation and subsequent dedifferentiation of vascular smooth muscle cells (VSMCs) are an absolute requirement for atherosclerosis and restenosis to occur after angioplasty. In cellular differentiation, proliferation, and apoptosis, miRNAs are thought to be a key regulator. In a VSMCs model of atherosclerosis, the expression of a novel miRNA, miR-18a-5p, and its downstream gene, syndecan4, were found to be of significance. In the early stages after balloon injury, miR-18a-5p was upregulated in differentiated VSMCs, while its expression decreased in dedifferentiated VSMCs. Thus, these results suggest that miR-18a-5p could modulate VSMC differentiation via the targeting of syndecan4 [77]. Smad2 promotes the differentiation of VSMCs [77]. Overexpression of syndecan4 has been to decrease the expression of Smad2, whereas its knockout results in upregulation of Smad2 expression in VSMCs. Eventually, we found that Smad2 promotes VSMC differentiation marker gene expression [77].

miRNAs serve as meaningful regulatory factors, participating in a series of pathophysiological cellular signaling pathways and molecular mechanisms controlling intricate atherosclerosis pathology [78, 79]. Mounting evidence has begun to explain clearly the significance of miRNAs in regulating pivotal signaling and lipid homeostasis pathways that alter the equilibrium of progression to regression of atherosclerotic plaques [80, 81]. However, these results suggest that expression levels of the miRNAs of the miR-17-92 cluster and HDAC9 genes in peripheral blood are not sufficient to act as biomarkers of high risk patients with unstable plaques compared to those that are stable [76].

In cardiovascular morphogenesis, the dysregulated expression of miR-17-92 leads to lethal cardiomyopathy, caused possibly in part by direct repression of PTEN and Cx43. Some results give prominence to the eminence of miR-17-92 being responsible for both normal and pathological effects of the heart and offer a useful platform to study novel antiarrhythmic therapeutics [10]. The miR-17-92 cluster may potentially modulate intricate biological processes; therefore, it has latent therapeutic applications as a novel drug for heart tissue and is likely to be more advanced than other therapies [82, 83].

### 5.3. The miR-17-92 Cluster in the Development of Bone

It has been increasingly discussed that miRNAs are a principal factor in osteoblast proliferation, differentiation, and apoptosis [84–86] (Table 1). The miR-17-92 gene cluster is widely present in vertebrates and has considerable involvement in the regulation of various biological processes. Compared with other related gene clusters, the miR-17-92 cluster is well conserved [12, 87] and has deeper involvement in various biological processes. These miRNA clusters exhibit inconsistent patterns of nucleotide differentiation not only between several animal species, but also between homologous miRNA genes [87]. To expound the possible direct role of miR-17-92 in bone cells, osteoblasts from miR-17-92 (+/delta) mice were cultured* in vitro*. The results suggest that miR-17, miR-92a, and miR-20a are highly expressed in bone tissue and osteoblasts. The expression levels of miR-17-92 were downregulated in osteoblast differentiation, the lowest expression observed in mature osteoblasts. In general, the miR-17-92 cluster exerts strict regulation of bone metabolism, principally in osteoblasts [12]. Consistent with this report, in order to identify the role of the miR-17-92 cluster in incisor tooth phenotype, conditionally knocking it out in type I collagen-expressing cells and evaluating by micro-CT and bone strength finite element analysis at weeks 5 and 12 demonstrated a 23 to 30% reduction in tissue and bone volume in the incisor teeth of conditional knockout (CKO) mice [12, 86, 88] (Table 1). The incisor teeth of CKO mice possessed 18-40% less stiffness and failure load compared to wild-type mice, due, as expected, to the reduced volume of bone and tissue. More interestingly, CKO mice were more sensitive to tooth movement because of a reduction in alveolar bone width and increased periodontal ligament space. Furthermore, the knockout of miR-17-92 in osteoclasts partially resulted in their activation with loss of trabecular bone by inhibition of the downregulation of miR-17-mediated protein-tyrosine phosphatase-OC in mice [88]. Taken together, this evidence suggests that the miR-17-92 cluster is essential for the development and maintenance of teeth strength by modulating the size of the teeth [86] (Figure 1) (Table 1). More specifically, we expect that miR-17-based treatments of osteoporosis and related bone wasting diseases will shortly be put into practice [88].

### 5.4. The miR-17-92 Cluster in Tumors

**Figure 3:**
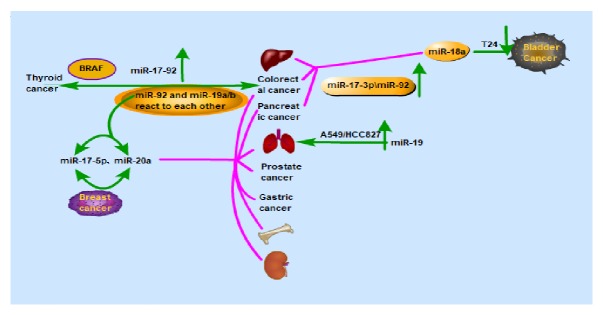
The miR-17-92 gene cluster is involved in the development of various tumors and performs a role in tumor suppression and promotion of cancer in different tumors. The members of this gene cluster have varying functions in the same or different tumors.

miRNAs are a large family of posttranscriptional regulators of gene expression that control cellular and developmental processes through the targeting of mRNA. These small noncoding RNAs (ncRNAs) are aberrantly expressed in cancer and are known to contribute to tumorigenesis and disease progression [89] (Table 1 and Figure 3). The miR-17-92 cluster, a known oncogene, is an indispensable factor in the development of cancer (Figure 1 and Figure 3). The individual mature miRNAs, such as miR-20a and paralog miR-106a, have a key role in glioma stem cell invasion of GSCs and may be a target for therapeutic strategies of glioblastoma [90].

The miR-17-92 cluster has been among the most widely reported miRNA clusters of late. It involves mickle biological processes as cell differentiation, proliferation, apoptosis, metastasis, and metabolism and is especially related to tumorigenesis. It is now apparent that the expression of the miR-17-92 cluster is increased in a wide spectrum of tumor cells and cancer types and so the cluster has been regarded as a potential oncogene in both mouse and human cells [91], (Table 1) and a representative oncogenic polycistronic miRNA. The miR-17-92 cluster promotes tumor development by regulating three different signaling pathways. Firstly, in some tumors, miR-17-92 represses the tumor suppressor gene, suppressor of cytokine signaling-1 (SOCS-1), in addition to stimulating the Janus kinase/signal transducer and activator of transcription (JAK-STAT) pathway which result in promotion of cell proliferation and inhibition of cell apoptosis. Secondly, the phosphoinositide-3 kinase (PI3K)/AKT/mTOR pathway is also a major considerable axis that inhibits apoptosis and activates antiapoptotic factors to regulate the development of the tumor, thereby promoting cell survival. Once activated, AKT regulates various forms of cellular activity such as proliferation, growth, and survival via the phosphorylation of diverse downstream targets. This pathway may downregulate tumor suppressor p53 gene expression, thus inhibiting apoptosis. Thirdly, PTEN, a phosphatase of phosphatidylinositol-trisphosphate, acts as a tumor suppressor to promote apoptosis by downregulation of the PI3K/AKT signaling pathway. The miR-17-92 cluster downregulates the expression of PTEN that activates the AKT/glycogen synthase kinase pathway and enhances proliferation of the cell and angiogenesis through the PI3K/AKT signaling pathway [91]. The miR-17-92 cluster is regarded as a promising new diagnostic and prognostic biomarker and canonical therapeutic in various domains not only in tumors.

## 6. Future Perspectives

Collectively, the members of the miR-17-92 cluster are of great importance in nerve repair and protection after strokes, heart disease, development of bone, and progression of cancer. The cluster's integration of transcriptional and posttranscriptional regulatory mechanisms is a significant process of biogenesis. In spite of the precise role of the miR-17-92 cluster in cardiovascular disease, osteoarthropathy, and development of tumors not being entirely clear, studies have revealed its antitumor function and the potential of targeting miR-17-92 components therapeutically both* in vitro *and* in vivo*. Although some functions and mechanisms are not yet fully understood, the value of the latest research in miRNAs undoubtedly lies in the understanding of the development of diseases and potential targets for their treatment. There are still many issues to be clarified, with many research avenues limited to animal models, clinical reliability requiring additional insight for the development of routine medical therapies. As the biological functions and molecular mechanisms are gradually revealed, our results may help identify novel biomarkers and molecular targets to treat patients in the future. We forecast that the prevention and treatment of human pathology based on miRNA biology will have broader application in the near future.

## Figures and Tables

**Table 1:** The roles of the miR-17-92 cluster in different diseases.

Diseases	Function	References
Neurological Diseases	mediating adult neural progenitor cells proliferation and differentiation	[67]
	ameliorating neurogenesis and angiogenesis	[67]
	reducing anxiety- and depression-related behaviors	[68]
	remodeling the functional recovery of nerve	[37, 69]

Heart Disease	regulating cardiac proliferation	[36]
	leading to cardiomyocyte proliferation in embryonic, postnatal, and even adult hearts	[36]
	reversing cardiac dysfunction and cardiac fibrosis	[36]
	regulating the physiological and pathophysiological processes of atherosclerosis	[76]
	partaking the vulnerable carotid plaques activities such as inflammation, apoptosis, and angiogenesis	[76]
	preventing the occurrence of cardiomyopathy	[10]

Development of Bone	stimulating osteoblast proliferation, differentiation, and apoptosis	[84–86]
	accelerating bone metabolism	[12]
	reducing tissue volume and bone volume	[12, 86, 88]
	increasing alveolar bone width and decreasing of periodontal ligament space	[86]
	lessening the loss of trabecular bone	[86]

Tumors	causing tumorigenesis	[89]
	inhibiting tumor suppressor genes	[91]

## Abbreviations

miRNA: MicroRNA
mRNA: Messenger RNA
AKT: Protein kinase B
mTOR: Mammalian target of rapamycin
PTEN: Phosphatase and tensin
CTGF: Connective tissue growth factor
TSP-1: Thrombospondin-1
KSHV: Kaposi's sarcoma-associated herpesvirus
EVI1: Ecotropic virus integration site 1
Shh: Sonic hedgehog
Fog2: Friend of Gata-2
CAD: Coronary artery disease
I/R: Ischemia/reperfusion
OS: Osteosarcomas
ASK1: Apoptosis signal-regulating kinase 1
APP: Amyloid precursor protein
CCND1: Cyclin D1
MAPK9: Mitogen-activated protein kinase 9
LATS2: Large tumor suppressor kinase 2
IRF: Interferon regulatory factor
JNK2: c-Jun amino terminal kinase 2
MAPK9: Mitogen-activated protein kinase 9
PKD2: Pathogenetic gene 2
GAB1: Grb2 anchoring protein 1
RBL1: Rhamnolipid 1
TSG101: Tumor sensitive gene 101
STAT3: Signal transduction, signal transduction transcriptional activator 3
TGF-*β*: Transforming growth factor-*β*
HIF-1*α*: Hypoxia inducible factor-1*α*
SVZ: Subventricular zone
JNK: c-Jun N-terminal kinase
MKK4: Mitogen-activated protein kinase kinase 4
BMP: Bone morphogenetic protein
BMPRII: Bone morphogenetic protein receptor II
FGFR1: Fibroblast growth factor receptor 1
Sgk1: Serum- and glucocorticoid-inducible protein kinase 1
ESC: Embryonic stem cell
LMC-MNs: Limb-innervating lateral motor column motor neurons
MNs: Motor neurons
ERK1/ERK2: Extracellular signal-regulated kinases 1 and 2
AMPK: AMP-activated protein kinase
MI: Myocardial cell ischemic injury
H_2_S: Hydrogen sulfide
ER: Endoplasmic reticulum
GRP78: Glucose regulatory protein 78
VSMCs: Vascular smooth muscle cells
CKO: Conditional knockout
ncRNAs: Noncoding RNAs
SOCS-1: Suppressors of cytokine signaling-1
JAK-STAT: Janus kinase/signal transducer and activator of transcription.
